# Estimates of genomic heritability and genome-wide association study for fatty acids profile in Santa Inês sheep

**DOI:** 10.1186/s12864-018-4777-8

**Published:** 2018-05-21

**Authors:** G. A. Rovadoscki, S. F. N. Pertile, A. B. Alvarenga, A. S. M. Cesar, F. Pértille, J. Petrini, V. Franzo, W. V. B. Soares, G. Morota, M. L. Spangler, L. F. B. Pinto, G. G. P. Carvalho, D. P. D. Lanna, L. L. Coutinho, G. B. Mourão

**Affiliations:** 1 Department of Animal Science, University of São Paulo (USP) / Luiz de Queiroz College of Agriculture (ESALQ), Av. Pádua Dias, 11, ESALQ/USP, Piracicaba, São Paulo 13418-900 Brazil; 2 Institute of Zootechny (IZ), Nova Odessa, SP Brazil; 3 Department of Animal Science, University of Nebraska, Lincoln, NE USA; 4 Department of Animal Science, Federal University of Bahia (UFBA), Salvador, BA Brazil

**Keywords:** Candidate genes, Fatty acid composition, Variance components, Ovine

## Abstract

**Background:**

Despite the health concerns and nutritional importance of fatty acids, there is a relative paucity of studies in the literature that report genetic or genomic parameters, especially in the case of sheep populations. To investigate the genetic architecture of fatty acid composition of sheep, we conducted genome-wide association studies (GWAS) and estimated genomic heritabilities for fatty acid profile in *Longissimus dorsi* muscle of 216 male sheep.

**Results:**

Genomic heritability estimates for fatty acid content ranged from 0.25 to 0.46, indicating that substantial genetic variation exists for the evaluated traits. Therefore, it is possible to alter fatty acid profiles through selection. Twenty-seven genomic regions of 10 adjacent SNPs associated with fatty acids composition were identified on chromosomes 1, 2, 3, 5, 8, 12, 14, 15, 16, 17, and 18, each explaining ≥0.30% of the additive genetic variance. Twenty-three genes supporting the understanding of genetic mechanisms of fat composition in sheep were identified in these regions, such as *DGAT2*, *TRHDE*, *TPH2*, *ME1*, *C6*, *C7*, *UBE3D*, *PARP14,* and *MRPS30.*

**Conclusions:**

Estimates of genomic heritabilities and elucidating important genomic regions can contribute to a better understanding of the genetic control of fatty acid deposition and improve the selection strategies to enhance meat quality and health attributes.

## Background

In recent years there has been a growing concern relative to the health attributes of foods that are consumed by increasingly health conscious consumers [1]. In particular, consumers are becoming gradually concerned relative to the amount of fatty acids in red meat [2]. Meat produced by ruminants is generally related to higher levels of saturated fatty acids (SFA), which are widely associated with the development of heart disease, stroke, diabetes, and obesity [3–5].

On the other hand, moderate levels of consumption of monounsaturated fatty acids (MUFA) are related to a decrease in serum cholesterol, consequently reducing the risk of heart diseases and strokes [4, 6–8]. Although found in a smaller proportion, red meat is also composed of polyunsaturated fatty acids (PUFA), which are strictly essential because they are not synthesized by humans and thus must be consumed daily to maintain proper body function [9]. These fatty acids influence several metabolic functions such as cell signaling, enzymatic regulation, eicosanoid synthesis, regulation of neuronal migration, neuromodulatory activity, and neurotransmitter activity [10, 11].

Despite the health concerns and nutritional importance of fatty acids, there is a relative paucity of studies in the literature that report estimates of genetic parameters, especially in the case of sheep populations. Estimating genetic parameters such as heritability, understanding the mechanisms underlying genetic variation in phenotypes, and predicting the genetic merit of an animal as a parent are fundamental pieces of information to enable genetic and ultimately phenotypic changes for designing successful animal breeding programs.

Fatty acids are complex traits, with several factors affecting their composition, such as sex, diet, age, and genetics [12]. Furthermore, routine phenotypic data collection is not practical given live-animal proxies do not exist for these traits and the expenses associated with collecting them. Thus, genomic information could play a critical role in enabling selection to improve them by allowing the design of animal breeding programs to increase the frequency of favorable alleles in the population [13, 14]. Concomitantly, the use of genomic information can increase the accuracy of estimated breeding values (EBV), thus increasing the rate of genetic change [15–17].

An important first step for the genetic evaluation of fatty acids content in meat is to investigate the genetic architecture of these complex traits and to identify variants associated with genes or regulatory elements through a genome-wide association study (GWAS) [18]. By estimating the degree to which these traits are heritable, and elucidating candidate genes, it could be possible to enable selection that will add commercial value to the sheep meat by providing consumers with a quality product that is also beneficial to health. Thus, the objectives of this study were to estimate genomic heritabilities and, for the first time, identify regions and candidate genes associated with fatty acid profiles in the *Longissimus dorsi* muscle of sheep using bivariate models.

## Results and discussion

### Fatty acid profiles

Descriptive statistics of the traits evaluated in this study are described in Table 1. The percentage of intramuscular fat (IMF) ranged between 1.62 and 4.93. These values were similar to those reported in sheep by [19–21], but lower than the values reported by [22, 23].

**Table 1:** Descriptive statistics for intramuscular fat and fatty acid (FA) profile traits of *Longissimus dorsi* muscle in Santa Inês sheep

Trait	Nomenclature	^a^N	Mean	^b^SD	^c^Min	^d^Max
Intramuscular fat (%)	IMF	216	3.46	0.769	1.62	4.93
Myristic acid (mg/mg)	C14:0	216	2.21	0.752	0.96	4.35
Palmitic acid	C16:0	216	22.17	1.949	10.51	29.43
Stearic acid	C18:0	216	20.29	3.342	12.53	33.26
Palmitoleic acid	C16:1	216	1.54	0.245	0.98	2.16
Oleic acid	C18:1	216	36.04	3.04	23.92	45.99
Linoleic acid	C18:2 ω6	216	3.99	1.151	1.47	8.05
Conjugated linoleic acid	CLA c9t11	216	0.43	0.184	0.076	0.924
Alpha-Linolenic acid	C18:3 ω3	216	0.30	0.150	0.00	0.797
Sum of saturated FA	SFAt	216	46.92	4.399	28.38	59.88
Sum of monounsaturated FA	MUFAt	216	45.17	3.919	32.02	62.21
Sum of polyunsaturated FA	PUFAt	216	6.55	1.927	2.37	12.39
Sum of omega-3 FA	ω3t	216	0.89	0.532	0.111	2.99
Sum of omega-6 FA	ω6t	216	5.23	1.618	1.82	10.6
Ratio of PUFA to SFA	PUFA/SFA	216	0.14	0.049	0.048	0.322
Ratio of ω6 to ω3	ω6/ω3	216	7.62	5.33	2.82	55.32

^a^number of phenotyped animals; ^b^standard deviation; ^c^minimum; ^d^maximum

The predominant individual SFAs were palmitic (C16:0, 22.17%), stearic (C18:0, 20.29%), and myristic (C14:0, 2.21%) corresponding to about 95% of the SFA present in the *Longissimus dorsi* muscle. These results agreed with those reported in previous studies [19, 23–25], where these SFAs were predominant in sheep. The C14:0 and C16:0 acids have been associated with increased serum level of cholesterol and LDL (low-density lipoproteins) and decreased levels of HDL (high density lipoproteins) in the blood, major factors related to obesity, atherosclerosis, hypertension, heart, and coronary diseases [3–5]. Although C18:0 is abundant in sheep meat, studies suggest that it has no impact on the increase of serum cholesterol level, being considered a neutral fatty acid relative to human health [26–28]. Additionally, a large part of the C18:1 content is produced by the desaturation of C18:0 [12].

In this study, the most abundant individual fatty acid was oleic (C18:1, 36.04%), corresponding to about 90% of MUFA, whereas the palmitoleic acid (C16:1, 2.21%) corresponded approximately only 5% of MUFA. These results are similar to those reported in previous studies [19, 20, 22, 24, 25]. C18:1 is one of the main fatty acids in the Mediterranean diet, where several studies report a low incidence of heart disease, despite high-fat consumption [29–31]. Similar to C18:1, C16:1 has been also associated with several health benefits, however, a recent study performed by Hoffmann et al. [8] suggested that increased consumption of C16:1 is associated with some diseases such as the development of cardiomyopathy.

In this study, PUFA concentrations were only 6.55%, the lowest among fatty acid groups. Consequently, a lower ratio of PUFA/SFA (0.14) was observed in this study, consistent with the range between 0.04 and 0.14 reported in the literature [20–24]. A diet with a low PUFA/SFA ratio is associated with a high level of serum cholesterol and strongly correlated with coronary diseases [32, 33]. The value recommended by the World Health Organization [34] is above 0.45, which is related to a lower incidence of coronary heart disease [35].

In this study, the parameters ω3t (sum of omega-3 fatty acids) and ω6t (sum of omega-6 fatty acids) corresponded to approximately 14 and 86% of the PUFA, respectively. The percentage for ω3t were lower than those reported in earlier studies [19, 21, 25], whereas for ω6t the percentage described by the same authors ranged from 3.49 to 12.65. For individual PUFA, the most abundant was linoleic acid (C18:2 ω6, 3.99%), which corresponded to approximately 61% of PUFA and made up about 77% of ω6 fatty acids. The concentrations of ω3t fatty acids for this study were low, less than 1% of the total fatty acids. Alpha-linolenic acid (C18:3 ω3) had the highest concentration, making up 37% of the ω3t fatty acids; however, only 0.37% of the total fatty acids. In previous studies the values for C18:2 ω6 ranged between 0.28 and 9.32%, while for C18:3 ω3 ranged between 0.42 and 2.03% [19–25].

Conjugated linoleic acid (CLA c9t11) corresponded to about 7% of the PUFA, and only 0.43% of the total fatty acids. Results ranging from 0.44 to 1.37 were observed in other ovine studies [19, 21, 23]. CLA c9t11 is a PUFA considered to be beneficial to human health given there is evidence that it has immunostimulatory, antioxidant, antimutagenic, anticarcinogenic, and anti-inflammatory action [36–38]. Due to the results observed in this study, a high ω6/ω3 ratio (7.62) was observed. A very high ω6/ω3 ratio stimulates the pathogenesis of many illnesses, including cardiovascular disease, cancer, and inflammatory and autoimmune diseases, whereas a lower ω6/ω3 ratio, below 4.0, exerts suppressive effects [39].

### Genomic heritability

Estimates of genomic heritability for fatty acids traits from the current study were between 0.25 and 0.46 (Table 2). Estimated genomic heritabilities for the individual SFA were high for C14:0 (0.44) and C18:0 (0.30), and moderate for C16:0 (0.25). For Scottish Blackface sheep, Karamichou et al. [40] using the traditional relationship matrix derived from pedigree information, reported slightly lower heritability estimates when compared to the current study for C16:0 (0.29), C18:0 (0.24), and C14:0 (0.14). Bolormaa et al. [41] also found lower genomic heritabilities for C14:0 (0.15), C16:0 (0.11), and C18:0 (0.19) in nine sheep breeds.

**Table 2:** Genomic heritabilities (h^2^) for fatty acid (FA) profile of *Longissimus dorsi* muscle in Santa Inês sheep

Trait	Nomenclature	^a^Vg	^b^Ve	^c^Vp	h^2^ (^d^SE)
Myristic acid	C14:0	0.11	0.13	0.24	0.44 (0.045)
Palmitic acid	C16:0	0.95	2.84	3.79	0.25 (0.033)
Stearic acid	C18:0	2.63	6.18	8.81	0.30 (0.037)
Palmitoleic acid	C16:1	0.014	0.034	0.048	0.30 (0.035)
Oleic acid	C18:1	2.23	5.77	8.00	0.28 (0.035)
Linoleic acid	C18:2 ω6	0.32	0.88	1.20	0.27 (0.034)
Conjugated linoleic acid	CLA c9t11	0.005	0.010	0.015	0.34 (0.045)
Alpha-Linolenic acid	C18:3 ω3	0.006	0.007	0.013	0.46 (0.045)
Sum of saturated FA	SFAt	4.45	9.44	13.89	0.32 (0.039)
Sum of monounsaturated FA	MUFAt	3.58	7.98	11.56	0.31 (0.038)
Sum of polyunsaturated FA	PUFAt	0.90	2.35	3.25	0.28 (0.034)
Sum of omega-3 FA	ω3t	0.05	0.09	0.15	0.37 (0.045)
Sum of omega-6 FA	ω6t	0.64	1.77	2.41	0.27 (0.034)
Ratio of PUFA to SFA	PUFA/SFA	0.0006	0.0015	0.0021	0.28 (0.034)
Ratio of ω6 to ω3	ω6/ω3	6.48	13.27	19.75	0.33 (0.042)

^a^additive genetic variance; ^b^ residual variance; ^c^ total phenotypic variance; ^d^ standard error

Moderate genomic heritability estimates for C16:1 (0.30) and C18:1 (0.28) acids were obtained in the present study. These results are almost identical to those reported by Karamichou et al. [40], 0.31 and 0.27, for the respective MUFA. Estimates of genomic heritability were high for CLA c9t11 (0.46), and moderate for C18:3 ω3 (0.34) and C18:2 ω6 (0.27). The heritabilities estimated by Karamichou et al. [40] was identical for C18:3 ω3 (0.34), however, were lower for CLA c9t11 (0.30) and C18:2 ω6 (0.10). Bolormaa et al. [41] estimated genomic heritability of 0.15 for C18:2 ω6 and Mortimer et al. [42], using the traditional relationship matrix, reported a heritability estimate of 0.22 for the same acid for crossbred and Merino sheep.

Estimates of genomic heritability reported herein for SFAt (0.32), MUFAt (0.31), and PUFAt (0.28) were all moderate. These results are lower than those reported by Karamichou et al. [40] who reported heritabilities above 0.40 for the same traits. Generally, these results suggest that there is an important genetic effect associated with phenotypic variation in these traits, although two different breeds were used between the current study and that of Karamichou et al. [40].

Estimates of genomic heritability for ω6t (0.27) and ω3t (0.37) were moderate. Genomic heritability estimates for these traits have not been previously reported in sheep populations. However, in beef cattle, genomic heritability estimates for ω3t and ω6t ranged from 0.08 to 0.34, slightly lower in general than the present study [2, 43–45].

The genomic heritability estimates for ω6/ω3 (0.33) and PUFA/SFA (0.28) ratios were moderate. These genetic parameter estimates have not been reported for a sheep population in the literature until now. In cattle for the ω6/ω3 and PUFA/SFA ratios, smaller genomic heritabilities were estimated for *Bos indicus* cattle, ranging from 0.07 to 0.14 [43, 44], and higher for *Bos taurus*, ranging from 0.12 to 0.42 [2, 45].

In general, genomic heritability estimates for the fatty acid content indicate that substantial genetic variation exists for the evaluated traits, thus, there is a possibility of altering the fatty acid profiles through selection. These results are important for sheep breeding programs that aim at improving the meat fatty acid composition. Although the genomic heritabilities of fatty acids are of different magnitude compared to the literature, it was possible to observe some consistency in the results, where higher estimates of genomic heritabilities were always obtained for C14:0, C16:0, C18:0, C16:1 and C18:1.

The moderate to high genomic heritabilities observed in this study can be attributed to the fact that there has been no direct selection for these traits in the current population, and thus substantial genetic variation has been maintained [46].

### Genome-wide association studies

The GWAS was performed to identify genomic regions (genomic windows) that explained the highest proportion of genetic variance. Each window was further divided into 10 continuous SNPs. A total of 27 different QTL regions ranging from 321,411 to 860,291 Kb were distributed in 11 different chromosomes: 1, 2, 3, 5, 8, 12, 14, 15, 16, 17, and 18. Twenty-three different putative candidate genes (PCG) harbored into these 11 associated genomic regions were identified, which are related to biological processes associated with fatty acid content in skeletal muscle.

### Saturated fatty acids

**Fig. 1:**
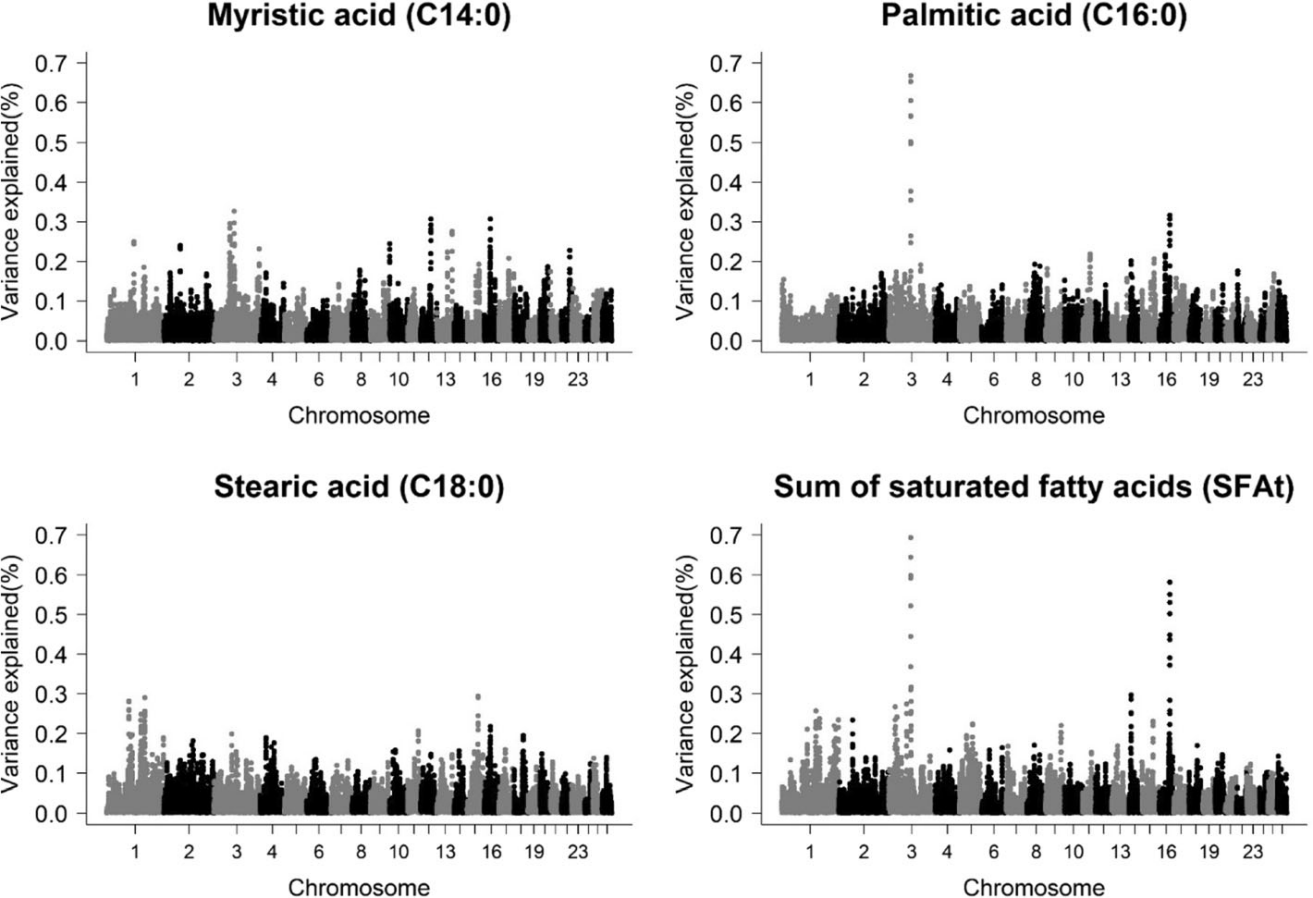
Manhattan plot for the proportion of genetic variance explained by windows of 10 adjacent SNPs for saturated fatty acids in the *Longissimus dorsi* muscle of Santa Inês sheep

Ten different QTL regions were associated with C14:0, C16:0, C18:0 and SFAt, which explained between 0.30 and 0.74% of the additive genetic variance (Table 3 and Fig. 1). QTL regions on chromosomes 3 and 16 were associated with C14:0, harboring *NPAS2* (neuronal PAS domain protein 2) and *MRPS30* (mitochondrial ribosomal protein S30) genes, respectively. *NPAS2* gene can be considered as PCG due to the important role in metabolic pathways for regulating lipid metabolism. These pathways include peroxisome proliferator-activated receptor alpha (PPARα), which acts in the control of beta-oxidation of fatty acids [47]. PPARs are receptors for nuclear hormones and transcription factors that regulate the expression of genes involved in lipid and glucose metabolism [48].

**Table 3:** Genomic regions and candidate genes associated with the saturated fatty acid (SFA) profile of the *Longissimus dorsi* muscle of Santa Inês sheep

Trait	Nomenclature	Genomic Window	Length (Kb)	^a^Vg (%)	Candidate genes
Myristic acid	C14:0	3:78397685–78915234	517,549	0.30	–
		3:99901070–100285876	384,806	0.33	*NPAS2*
		16:30002131–30349083	346,952	0.31	*MRPS30*
Palmitic acid	C16:0	3:107748212–108088562	340,350	0.67	*TPH2*, *TRHDE*
		16:55961351–56458897	497,546	0.32	*CDH12*
Stearic acid	C18:0	1:185195800–185614760	418,960	0.30	*PARP14*
		15:52854077–53676145	822,068	0.39	*DGAT2*, *WNT11*
Sum of SFA	SFAt	3:107809276–108146152	336,876	0.74	*TPH2*, *TRHDE*
		3:109215221–109694971	479,750	0.32	–
		14:11458148–11862331	404,183	0.30	–
		15:52854077–53676145	822,068	0.58	*DGAT2*, *WNT11*

^a^additive genetic variance explained by each window

*MRPS30* is related to the synthesis of proteins inside the mitochondria, being one of more than 70 mitochondrial ribosomal protein components that are encoded by the nuclear genome [49]. One of the Gene Ontology (GO) terms related to this gene is cellular apoptosis (GO:0006915), playing an important role in the induction of apoptosis by saturated fatty acids in several cells [6, 8, 50–52].

Two genomic regions were associated with C16:0 content, on chromosome 3 at 107.7 Mb. In the first region two PCG were identified, *TPH2* (tryptophan hydroxylase 2) and *TRHDE* (thyrotropin releasing hormone degrading enzyme). *TPH2* is associated with the serotonergic system, involved in a large number of physiological functions including lipolysis [53, 54] and oxidoreductase activity (GO:0004510), which is involved in oxidation reactions of a variety of substrates including retinoids and steroids [55].

*TRHDE* gene is related to thyrotropin-releasing hormone in humans [56]. In general, thyroid hormone plays a critical role in mediating changes in development and metabolism in humans [57, 58]. GO annotations relate this gene to the integral component of the plasma membrane (GO:0005887). In sheep, the *TRHDE* gene is an important candidate gene, because it was related to the amount of internal fat in Merino sheep [59], and post-weaning gain in three sheep populations [60].

In the region on chromosome 16 at 55 Mb, the *CDH12* (cadherin 12) gene was found, which was associated with the C14:0 and C18:0 content in milk of Holstein cows and with the Wnt signaling pathway [61]. The Wnt signaling pathway has been shown to have inhibitory effects of adipogenesis [62, 63]. Wnt signaling pathways dysfunctions are associated with obesity and lipodystrophy [64, 65]. Additionally, GO annotations relate the *CDH12* as an integral component of the cell membrane (GO:0016021).

Two regions on different chromosomes were associated with C18:0, on chromosome 1 at 185 Mb and chromosome 15 at 52.85 Mb. Chromosome 1 harbors the *PARP14* gene (poly (ADP-ribose) polymerase family member 14) and chromosome 15 includes *DGAT2* (diacylglycerol o-acyltransferase 2) and *WNT11* (Wnt family member 11) genes. *PARP14* is involved in the aerobic glycolysis process and promotes the survival of cancer cells by regulating the transcription of template DNA (GO:0006355).

*DGAT2* (diacylglycerol o-acyltransferase 2), which is an important gene associated with the catalysis of the final stage of triacylglycerol biosynthesis [66] was identified in the region of chromosome 15 at 52.85 Mb. As the *CDH12* gene, *WNT11* gene is related to the Wnt signaling pathway and it is also linked to adipogenesis processes [67].

For SFAt, four genomic regions were found. Chromosome 3 at 107.8 Mb overlapped with the region associated with C16:0 (chromosome 3 at 107.7 Mb) and the same candidate genes (*TPH2* and *TRHDE*) were found. The region of chromosome 15 at 52.85 Mb was common to C18:0, therefore, the same candidate genes were observed (*DGAT2* and *WNT11*). This is expected since a high proportion of SFAt is composed by C16:0 and C18:0. In the regions of chromosome 3 at 109 Mb and chromosome 14 at 11 Mb, no candidate genes were identified.

### Monounsaturated fatty acids

**Fig. 2:**
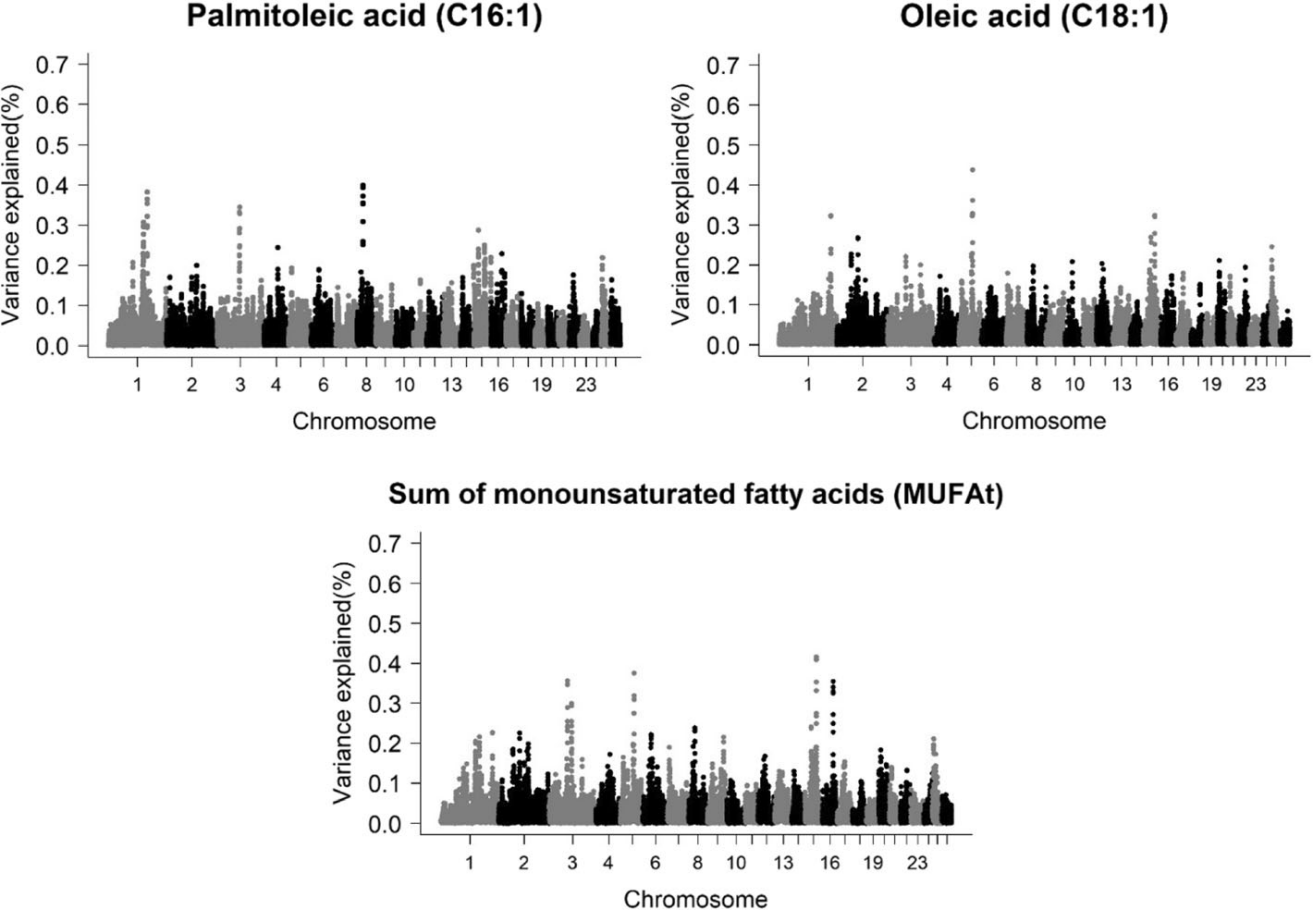
Manhattan plot for the proportion of genetic variance explained by windows of 10 adjacent SNPs for monounsaturated fatty acids in the *Longissimus dorsi* muscle of Santa Inês sheep

For MUFA, ten different genomic regions were found (Table 4 and Fig. 2). For C18:1, three genomic regions encompassing PCG were found. The first region was observed on chromosome 1 at 247 Mb and the PCG detected was *COPB2* (coatomer protein complex subunit beta 2). The *COPB2* gene plays an essential role in the Golgi membrane (GO:0000139) and metabolic pathways related to the transport of cholesterol and sphingolipid of the Golgi complex and the endoplasmic reticulum to the plasma membrane in the region (GO:0006888).

**Table 4:** Genomic regions and candidate genes associated with the monounsaturated fatty acids (MUFA) profile of the *Longissimus dorsi* muscle of Santa Inês sheep

Trait	Nomenclature	Genomic Window	Length (Kb)	^a^Vg (%)	Candidate genes
Oleic acid	C18:1	1:247008204–247578979	570,775	0.32	*COPB2*
		5:69713533–70306809	593,276	0.44	–
		15:52894279–53729913	835,634	0.32	*DGAT2*
Palmitoleic acid	C16:1	1:168328606–168777508	448,902	0.31	*ALCAM*
		1:185195800–185614760	418,960	0.38	*PARP14*
		3:107809276–108146152	336,876	0.33	*TPH2*, *TRHDE*
		3:109215221–109694971	479,750	0.34	–
		8:28651040–29263838	612,798	0.40	*FOXO3*, *OSTM1*
Sum of MUFA	MUFAt	3:109215221–109694971	479,750	0.30	–
		3:88827198–89234916	407,718	0.36	–
		5:69713533–70306809	593,276	0.38	–
		15:52894279–53729913	835,634	0.42	*DGAT2*

^a^additive genetic variance explained by each window

In the second region located on chromosome 5 at 69 Mb, no candidate genes were found. The region on chromosome 15 at 52.89 Mb overlapped with the C18:0 region (chromosome 15 at 52.85 Mb) including a common PCG (*DGAT2*). This genetic correlation can be attributed to the fact that C18:0 is a precursor of C18:1, by the desaturation process [68].

We found five genomic regions associated with C16:1 acid. On chromosome 1 at 168 Mb the *ALCAM* (activated leukocyte cell adhesion molecule) gene was found, which encodes the activated leukocyte adhesion molecule, linked to cell adhesion and migration (GO:0007155), adaptive immune response (GO:0002250), and immunological synapse (GO:0001772). Another region on chromosome 1 at 185 Mb was common between C18:0 and C16:1, so the same candidate gene was detected (*PARP14*). Two regions of chromosome 3 at 107.8 and 109 Mb associated with C16:1 were found, however in the second, no candidate genes were identified. The first region was also associated with SFAt and C16:0, thus the same candidate genes were identified (*TPH2* and *TRHDE*). Because C16:0 acid is a precursor of C16:1 (desaturation) and C18:0 (elongation) [68] and is related to the SFAt, it is expected that there are common genetic mechanisms for these fatty acids.

In the region of chromosome 8 at 28 Mb two candidate genes *FOXO3* (forkhead box O3) and *OSTM1* (osteopetrosis associated transmembrane protein 1) were associated with C16:1. The *FOXO3* gene is involved in functions related to apoptosis through the expression of genes necessary for cell death [69]. Unsaturated or saturated fatty acids play an essential role in cell apoptosis process, such as hepatocytes [70] and β-cells [71].

*OSTM1* encodes a protein involved in the degradation of G proteins via the ubiquitin-dependent proteasome pathway (RefSeq, Jul 2008 [72]). These proteins have been reported to be related to the regulation of body weight and metabolic function, hyperinsulinemia, glucose tolerance, and insulin resistance [73].

Four genomic regions associated with MUFAt were found, with two being in common to C18:1 (chromosome 5 at 69 Mb and chromosome 15 at 52.89 Mb), and one common to C16:1 (chromosome 3 at 109 Mb). Thus, PCG was only found on chromosome 1 (*DGAT2*). The levels of lipids in the blood plasma may have a direct influence on adaptive immunity [74]. Another region was observed on chromosome 3 at 88 Mb without any associated candidate gene.

### Polyunsaturated fatty acids

**Fig. 3:**
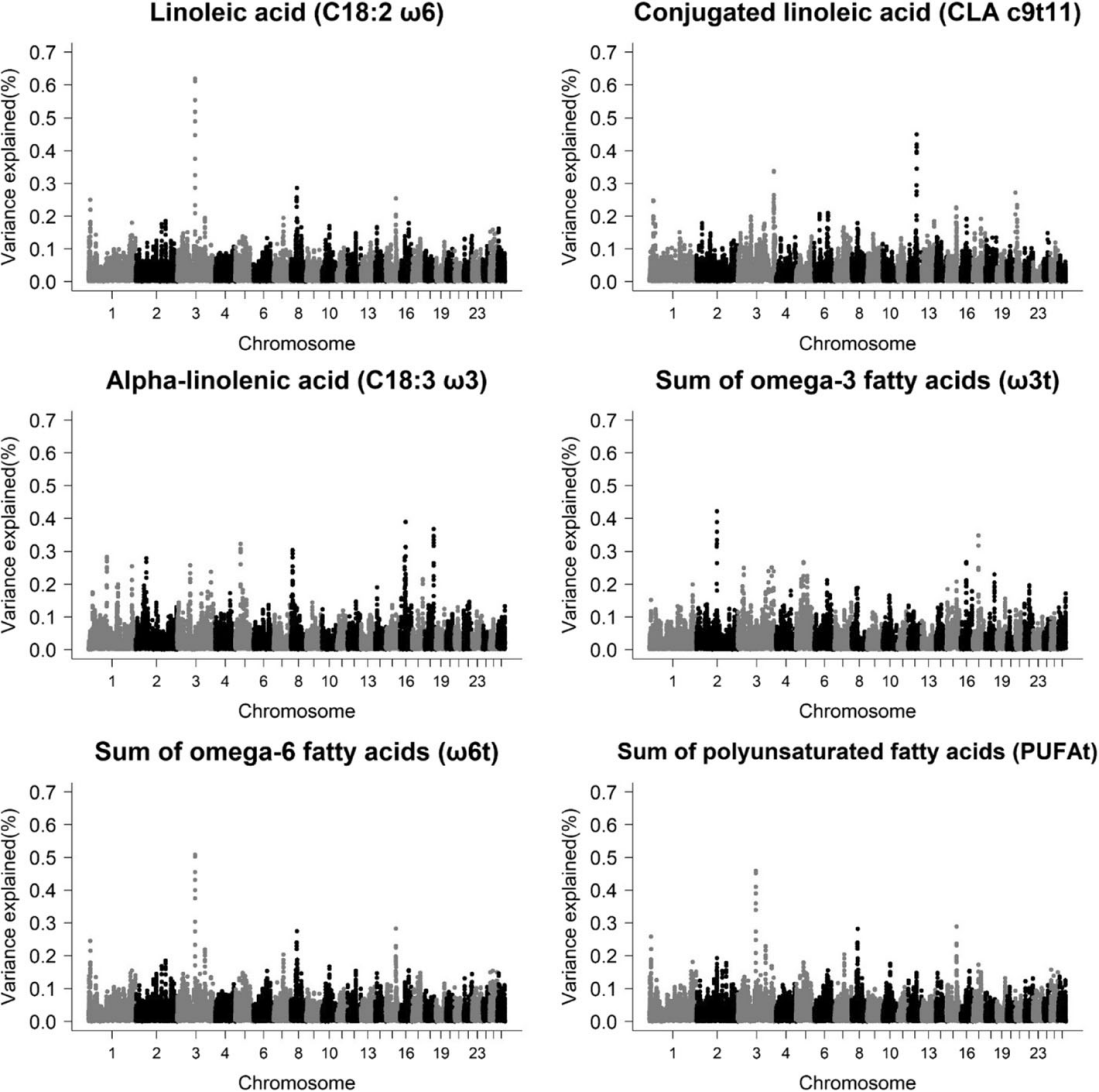
Manhattan plot for the proportion of genetic variance explained by windows of 10 adjacent SNPs for polyunsaturated fatty acids in the *Longissimus dorsi* muscle of Santa Inês sheep

Eleven different genomic regions were observed for C18:3 ω3, C18:2 ω6, CLA c9t11, ω6t, ω3t, and PUFAt, distributed over eight chromosomes (Table 5 and Fig. 3). For C18:2 ω6, two regions were found on different chromosomes. One of these regions was common to C16:1 and SFAt (chromosome 3 at 107.8 Mb) and overlapped to C16:0 (107.7). Thus, the same PCG were identified (*TPH2* and *THRDE*). In the region of chromosome 8 at 35 Mb, no PCG were found.

**Table 5:** Genomic regions and candidate genes associated with the polyunsaturated fatty (PUFA) acids profile of the *Longissimus dorsi* muscle of Santa Inês sheep

Trait	Nomenclature	Genomic Window	Length (Kb)	^a^Vg (%)	Candidate genes
Linoleic acid	C18:2 ω6	3:107809276–108146152	336,876	0.62	*TPH2*, *TRHDE*
		8:35511823–35833234	321,411	0.30	*–*
alpha-Linolenic acid	C18:3 ω3	5:35136857–35559907	423,050	0.32	*TNFAIP8*
		8:10032564–10601751	569,187	0.30	*UBE3D*, *ME1*
		16:32987534–33510366	522,832	0.39	*PLCXD3*, *C6*, *C7*
		18:55048766–55542400	493,634	0.37	*CCDC88C*, *FBLN5*
Conjugated linoleic acid	CLA c9t11	3:212363790–212692333	328,543	0.34	*CACNA1C*
		12:49048350–49840917	792,567	0.45	*–*
Sum of omega-3 FA	ω3t	2:122297721–123158012	860,291	0.42	–
		16:33207525–33550464	342,939	0.30	*C6*, *C7*
Sum of omega-6 FA	ω6t	3:107809276–108146152	336,876	0.51	*TPH2*, *TRHDE*
		15:58185924–58511281	325,357	0.30	–
Sum of PUFA	PUFAt	3:107809276–108146152	336,876	0.46	*TPH2*, *TRHDE*
		8:35511823–35833234	321,411	0.30	*–*
		15:58185924–58511281	325,357	0.30	–

^a^additive genetic variance explained by each window

For C18:3 ω3, four genomic regions were found on chromosomes 5, 8, 16, and 18. In the region of chromosome 5 at 35 Mb, the candidate gene identified was the *TNFAIP8* (tumor necrosis factor, alpha-induced protein 8). In humans, this gene has been confirmed to be important for the maintenance of immune homeostasis and a regulator of apoptosis, and plays a main role in the oncogenesis of several cancer types [75].

In the region located on chromosome 8 at 10 Mb the *ME1* (malic enzyme 1) and *UBE3D* (ubiquitin-protein ligase E3D) genes were identified. *ME1* is related to the tricarboxylate transport system that produces NADPH and acetyl-CoA, necessary components in fatty acid biosynthesis [76]. On the other hand, the *UBE3D* gene is related to E3 ubiquitin-protein ligase which accepts ubiquitin from specific E2 ubiquitin-conjugating enzymes, and transfers it to substrates, usually supporting their degradation by the proteasome [77, 78]. In a general context, the function of the ubiquitin-proteasome pathway can be controlled physiologically, in part, by fatty acids within cellular membranes [79].

On chromosome 16 at 32 Mb the following candidate genes associated with C18:3 ω3 were detected: *PLCXD3* (phosphatidylinositol-specific phospholipase C X domain containing 3), *C6* (complement C6), and *C7* (complement C7) genes. *PLCXD3* gene was related to phospholipases, a group of enzymes that hydrolyze the phospholipids in fatty acids and other lipophilic molecules (GO:0016042). *C6* and *C7* are genes related to the membrane attack complex (GO:0005579), playing key roles in the innate and acquired immune response (GO:0045087) by assisting in inflammatory responses against infections [80]. The ω3 acids influence the activation of inflammatory cells processes from signal transduction to protein expression, even involving effects at the genomic level [81].

In the region of chromosome 18 at 55 Mb, related to C18:3 ω3, the PCG were *CCDC88C* (coiled-coil domain containing 88C) and *FBLN5* (fibulin-5). *CCDC88C* has been described as a negative regulator of the Wnt signaling pathway (GO: 0016055). *FBLN5* plays an essential role in the assembly of elastic fibers that provide strength and flexibility to the connective tissue. This gene also exerts an important pleiotropic effect together with the *DGAT1* gene in Holstein dairy cows [82].

For CLA c9t11, two genomic regions were found in the region on chromosome 3 at 212 Mb, the PCG identified was the *CACNA1C* (calcium voltage-gated channel subunit alpha1 C). *CACNA1C* belongs to a family of genes that provide instructions for constructing calcium channels (GO:0005891). Long chain fatty acids are involved in the calcium channel activation processes, possibly acting at some nearby lipid binding sites on these channels or directly over the channel protein itself [83–85]. In the region of chromosome 12 at 49 Mb, no PCG related to CLA c9t11 was observed.

Two genomic regions were associated with ω3t on two different chromosomes. The region of chromosome 16 at 33 Mb overlapped with the genomic region associated with C18:3ω3 and the same genes were observed (C6 and C7). For ω6t two genomic regions were observed and explained greater than 0.30% of the additive genetic variance. One of these regions was common to C18:2 ω6 on chromosome 3 at 107 Mb, and the same candidate genes were found (*TPH2* and *TRHDE*). In the region of chromosome 15 at 58 Mb, none candidate gene was found. For PUFAt, three genomic regions in common to 18:2 ω6 and ω6t were found, consistent with the fact that the same regions were associated with other PUFA traits.

### ω6/ω3 and PUFA/SFA ratios

**Fig. 4:**
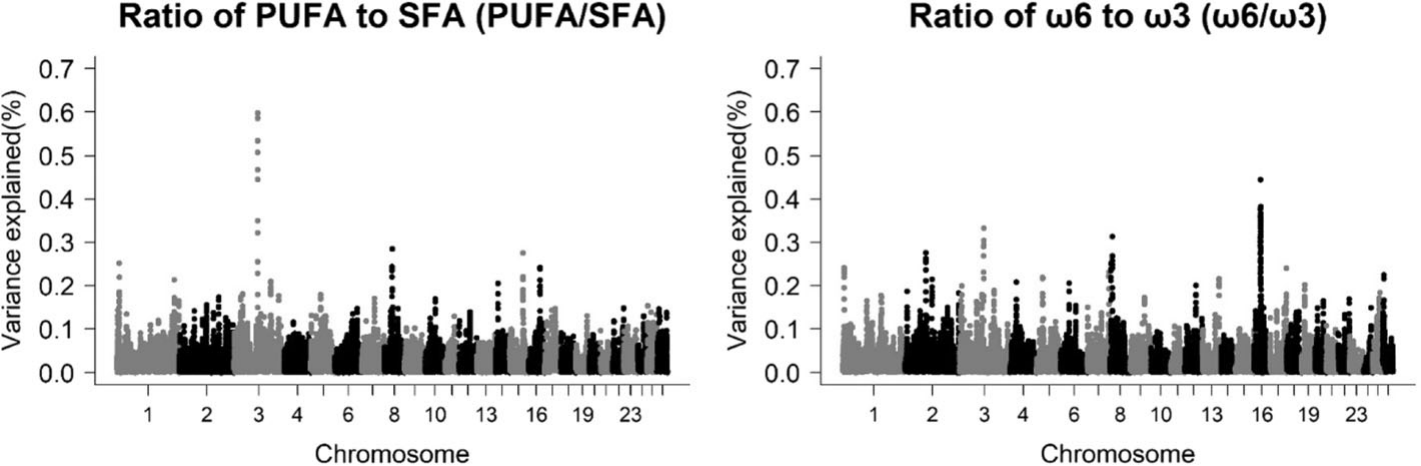
Manhattan plot for the proportion of genetic variance explained by windows of 10 adjacent SNPs for PUFA/SFA and ω6/ω3 ratios in the *Longissimus dorsi* muscle of Santa Inês sheep

Six genomic regions were observed for the ω6/ω3 ratio, whereas only one genomic region was observed on chromosome 3 for the PUFA/SFA ratio (Table 6 and Fig. 4). For the ω6/ω3 ratio, only the regions of chromosome 8 at 10 Mb and chromosome 16 at 30 Mb harbored PCG. The region of chromosome 8 was common to C18:3 ω3, thus, the same candidate genes were observed (*UBE3D* and *M1*). This result is expected since C18:3 ω3 is one of the most abundant ω3 acids, and consequently, it is directly involved in the ω6/ω3 ratio. The region on chromosome 16 was common to C14:0, so the same candidate gene was observed (*MRPS30*) suggesting that there may be a genetic correlation with some other polyunsaturated fatty acid that has not been addressed in this study. For the PUFA/SFA ratio, only one region on chromosome 3 at 107.8 Mb, common to several individual fatty acids and groups of the fatty acids (C16:0, C16:1, PUFAt, SFAt, C18:2 ω6, and ω6t) was found. This was expected given the PUFA/SFA ratio uses all these fatty acids for its calculation.

**Table 6:** Genomic regions and candidate genes associated with the ω6/ω3 and PUFA/SFA ratios of the *Longissimus dorsi* muscle of Santa Inês sheep

Trait	Nomenclature	Genomic Window	Length (Kb)	^a^Vg (%)	Candidate genes
Ratio of ω6 to ω3	ω6/ω3	3:109215221–109694971	479,750	0.33	–
		8:10032564–10601751	569,187	0.31	*UBE3D, ME1*
		16:28050501–28436992	386,491	0.38	–
		16:29245079–29691641	446,562	0.44	–
		16:30002131–30349083	346,952	0.38	*MRPS30*
Ratio of PUFA to SFA	PUFA/SFA	3:107809276–108146152	336,876	0.60	*TPH2, TRHDE*

^a^additive genetic variance explained by each window

In the present study, important genomic regions associated with fatty acids profile were identified, providing an improved biological understanding of fatty composition in ovine. The identification of these genes in sheep using genomic approach is the first step to search for causal variations of large effect and can contribute to genetic evaluations of relevant traits in the future. Despite the observed moderate genomic heritabilities, our results suggest the meat fatty acid profile of Santa Inês sheep is controlled mainly by many QTL of small effects. This was expected since from the quantitative genetics point of view, meat fatty acids profile is a complex trait controlled by multiple genes and is influenced by several loci throughout the genome [86]. Additionally, genomic regions identified explained most, but not all, of the additive genetic variance for traits, possibly because there are causal mutations with low minor allele frequency and consequently in incomplete linkage disequilibrium with the SNPs [86]. Therefore, the identification of genetic variants of large effects can be difficult because the contribution of each genomic region to additive genetic variance is small. This implies that genomic selection may be an essential tool for the improvement of these traits, since it captures the effects of all genetic markers simultaneously. In addition, these genomic regions can be used in fine mapping studies, which will be useful to search for causative variations.

## Conclusion

Moderate to high genomic heritabilities were estimated for fatty acid profiles in this study, suggesting that these traits can be altered by selection. Several genomic regions and PCG associated with fatty acid profiles were identified, which can be used in later studies to fine-mapping the causal variations. The results described in this study have not been previously reported in sheep. Thus, this research is the first step toward understanding the genetic and metabolic mechanisms involved in the phenotypic determination of fatty acids in sheep meat; information that can be useful to define the selection strategies for these traits with the aim to obtain a benefic product to human health.

## Methods

### Animals and phenotypes

The study was conducted using phenotypes of 216 non-castrated male Santa Inês sheep, selected randomly, with unknown pedigree. Genomic relationships revealed full- and half-sibling relationships as well as unrelated individuals, with an average relationship among individuals of − 0.005 (standard deviation = 0.0648; minimum = − 0.12; maximum = 0.97). The experiment was conducted at the Experimental Farm of the School of Veterinary Medicine and Animal Science belonging to the Federal University of Bahia, located in the municipality of São Gonçalo dos Campos – Bahia. Animals were raised in individual pens (1.0 m^2^) from 110 days of age on average and slaughtered around 90 days later. Meat samples were collected from the *Longissimus dorsi* muscle, between the 12 and 13th ribs of each animal, 24 h after slaughter and stored at − 20 °C until further analysis to determine the fatty acid profile and intramuscular fat percentage (IMF). The animals were raised in three different periods between 2015 and 2016. The concentrate for these 216 ovine consisted of ground corn grain, soybean meal, urea, mineral supplement specific for sheep and the forage was Tifton 85 hay. All diets consisted of 2.45 Mcal/kg DM of metabolisable energy, all of them formulated according to the NRC (2007), in order to meet the nutritional requirements for lambs with estimated weight gains of 200 g/day. Of these, 100 individuals were separated into 5 experimental groups with 20 animals each and received diets comprised of cottonseed (ground or whole) with presence or absence of chitosan (in five different levels). Animals were fed with a forage:concentrate ratio of 50:50.

Other group of 72 Santa Inês lambs were separated into four sets with 18 individuals each. The diets corresponded to two sizes of hay particles of Tifton-85 (6 and 13 mm) and two forage:concentrate ratios (50:50 and 70:30). The diets for these 172 animals were formulated to be isonitrogeneous with 16% crude protein.

Another set of animals consisted of 38 animals separated by four groups ranging from 9 to 10 animals each. Each group received diets with a forage:concentrate ratio of 50:50 with different levels of crude protein: fixed level with 11% crude protein; fixed level with 13% crude protein; oscillating level 11 and 13% crude protein; oscillating level 13 and 11% crude protein. The remaining six animals received the same diet based on hay and concentrate (50:50), but with 16% crude protein.

IMF content was determined at the Meat Science Laboratory in the Department of Animal Science at UFLA (Lavras, Minas Gerais, Brasil), performed by near-infrared spectrophotometer according to the AOAC: 2007–04 method [87] in *Longissimus dorsi* muscle (~ 180 g, without fat cover) using FoodScan™ (FOSS, Hillerød, Denmark) with an artificial neural network calibration model and database for the determination of intramuscular fat [88].

The extraction, methylation and reading steps for the determination of fatty acid composition in the *Longissimus dorsi* (30 g) muscle were conducted at the Animal Nutrition and Growth Laboratory at ESALQ (Piracicaba, São Paulo, Brazil). The fatty acid extraction was conducted as described by Hara and Radin [89]. Subsequently, extracted lipids were hydrolyzed and methylated as described by Christie [90]. Fatty acid methyl esters were quantified with a gas chromatograph (ThermoFinnigan, Thermo Electron Corp., MA, USA) equipped with a flame ionization detector and a 100 m Supelco SP-2560 (Supelco Inc., PA, USA) fused silica capillary column (100 m, 0.25 mm and 0.2 μ m film thickness). The column oven temperature was held at 70 °C for 4 min, then increased to 170 °C at a rate of 13 °C min ^− 1^, and subsequently increased to 250 °C at a rate of 35 °C min ^− 1^, and held at 250 °C for 5 min. The gas fluxes were 1.8 mL min ^− 1^ for carrier gas (He), 45 mL min ^− 1^ for make-up gas (N^2^), 40 mL min ^− 1^ for hydrogen, and 450 mL min ^− 1^ for synthetic flame gas. One μL of the esterified extract was injected in the chromatographer. Injector and detector temperatures were 250 and 300 °C, respectively.

The fatty acids were identified by comparison of the retention times of methyl esters in the samples with standards of fatty acids from butter reference BCR-CRM 164, Anhydrous Milk Fat-Producer (BCR Institute for Materials and Reference Measurements) and also with commercial standard for 37 fatty acids Supelco TM Component FAME Mix (cat 18,919, Supelco, Bellefonte, PA). Fatty acids were quantified by normalizing the areas of methyl esters. Fatty acids were expressed as a weight percentage (mg/mg), obtained using ChromQuest 4.1 software (Thermo Electron, Milan, Italy).

After extraction, 47 different fatty acids were obtained from the *Logissimus dorsi muscle* of Santa Inês sheep. Of these, the following fatty acids were selected: myristic (C14:0), palmitic (C16:0), stearic (C18:0), palmitoleic (C16:1), oleic (C18:1, cis-9), linoleic (C18: 2 ω6), conjugated linoleic acid (CLA c9t11) and alpha-linolenic (C18: 3 ω3). These individual fatty acids were selected considering their importance for human health and abundance in the samples evaluated. The sum of saturated acids (C6:0 + C8:0 + C10:0 + C11:0 + C12:0 + C13:0 + C13:0 anteiso + C13:0 iso + C14:0 + C14:0iso + C15:0 + C15:0 anteiso + C15:0 iso + C16:0 + C16:0 iso + C17:0 + C17:0 iso + C18:0 + C20:0 + C22:0 + C23:0 + C24:0), sum of monounsaturated acids (C10:1 + C14:1 c9 + C16:1 + C17:1 + C18:1 c11 + C18:1 c12 + C18:1 c13 + C18:1 c15 + C18:1 + C18:1 t16 + C18:1 t9 + C20:1 + C22:1 + C24:1), sum of poly-unsaturated fatty acids (C18:2 ω6 + CLA c9t11 + C18:3 ω3 + C18:3 ω6 + C20:3 ω6 + C20:2 ω6 + C20:3 ω3 + C20:4 ω6 + C20:5 ω3 + C22:5 ω3 + C22:6 ω3), sum of omega 6 acids (C18:2 ω6 + C18:3 ω6 + C20:3 ω6 + C20:2 ω6 + C20:4 ω6), and sum of omega 6 acids (C18:3 ω3 + C20:3 ω3 + C20:5 ω3 + C22:5 ω3 + C22:6 ω3) were calculated. We also calculated the polyunsaturated/saturated fatty acid and omega 6/omega 3 ratios.

### Genotyping of animals and quality control

Genotyping was performed at the Biotechnology Laboratory at ESALQ, Piracicaba, São Paulo, Brazil. A total of 216 animals were genotyped using 54,241 SNPs from the Ovine SNP50 BeadChip (Illumina Inc., San Diego, CA). Quality control of the SNPs consisted of excluding those located on sex chromosomes; monomorphic; minor allele frequency lower than 0.05; call rate lower than 90%; and Hardy-Weinberg equilibrium deviations (difference between expected and observed frequency of heterozygous) higher than 0.15. All genotypes achieved a call rate greater than 90%, and thus no genotypes were removed due to this threshold. After quality control, 42,363 SNPs and 216 animals were retained for further analyses.

### Genetic analysis

Genetic (co)variance components were estimated by restricted maximum likelihood (REML) method with an average information algorithm using a genomic relationship matrix, which was calculated as described by Van Raden [16]. Models that account for genomic information are powerful tools for capturing a large proportion of the additive genetic variation, increasing the estimation accuracy of the genetic parameters [2, 91]. The analyses were performed using the BLUPF90 family programs [92]. Genomic heritabilities and genomic breeding values (GEBV) for fatty acid profiles were estimated by bivariate analyses with IMF as an anchor trait. The variance components were fixed to the estimates obtained from the univariate analysis for IMF only, and the realized genomic relationship matrix calculated from the SNP marker information was fitted [16]. The anchor trait approach was used to minimize the bias associated with a sample of selected individuals [93] in the event that selection had occurred for some measure of fatness in this population. Additionally, it is expected that the use of multivariate models will have larger or at least similar power compared to univariate models [94, 95]. Use of related phenotypic traits can improve the power of candidate gene detection [96]. Furthermore, the pattern of pleiotropy could support the detection of candidate genes underlying an association and the genetic mechanisms responsible [41]. For the bivariate analyses the following genomic best linear unbiased prediction (GBLUP) model was used:

$$ \left[\begin{array}{c}{\boldsymbol{y}}_1\\ {}{\boldsymbol{y}}_2\end{array}\right]=\left[\begin{array}{cc}{\boldsymbol{X}}_1& 0\\ {}0& {\boldsymbol{X}}_2\end{array}\right]\left[\begin{array}{c}{\boldsymbol{b}}_1\\ {}{\boldsymbol{b}}_2\end{array}\right]+\left[\begin{array}{cc}{\boldsymbol{Z}}_1& 0\\ {}0& {\boldsymbol{Z}}_2\end{array}\right]\left[\begin{array}{c}{\boldsymbol{u}}_1\\ {}{\boldsymbol{u}}_2\end{array}\right]+\left[\begin{array}{c}{\boldsymbol{e}}_1\\ {}{\boldsymbol{e}}_2\end{array}\right] $$

where the vectors ***y***_**1**_ and ***y***_**2**_ refer to the observations of the IMF and fatty acid traits, respectively; ***X***_**1**_ and ***X***_**2**_ are the design matrices and ***b***_**1**_ and ***b***_**2**_ are the vectors of the fixed effects for the first and second trait, respectively; ***Z***_**1**_ and ***Z***_**2**_ are the design matrices and ***u***_**1**_ and ***u***_**2**_ are the vector of genomic breeding values of the two traits; and ***e***_**1**_ and ***e***_**2**_ are the vectors of the residual effects. For univariate and bivariate analyses the vector ***b*** included the fixed effect of contemporary groups (*n* = 14, comprised of 6 to 20 individuals) where formed by diet and period of confinement as previously reported. Also, it was assumed that $$ \left[\begin{array}{c}{\boldsymbol{u}}_{\mathbf{1}}\\ {}{\boldsymbol{u}}_{\mathbf{2}}\end{array}\right]\sim N\left(0,\boldsymbol{G}\bigotimes \boldsymbol{H}\right) $$, where ***G*** is the realized genomic relationship matrix and $$ \boldsymbol{H}=\left[\begin{array}{cc}{\sigma}_{u_1}^2& {\sigma}_{u_{12}}\\ {}{\sigma}_{u_{21}}& {\sigma}_{u_2}^2\end{array}\right] $$ is the variance and covariance matrix of the genomic breeding values for the two traits; and $$ \left[\begin{array}{c}{\boldsymbol{e}}_{\mathbf{1}}\\ {}{\boldsymbol{e}}_{\mathbf{2}}\end{array}\right]\sim N\left(0,\boldsymbol{I}\otimes \boldsymbol{R}\right) $$, where $$ \boldsymbol{R}=\left[\begin{array}{cc}{\sigma}_{e_1}^2& {\sigma}_{e_{12}}\\ {}{\sigma}_{e_{21}}& {\sigma}_{e_2}^2\end{array}\right] $$ is the residual variance and covariance matrix for the two traits.

For GWAS the effects of the SNPs ($\widehat{a}$) were obtained from GEBV using the following equation described by [97]:

$$ \widehat{a}={DW}^{'}{\left[{WDW}^{'}\right]}^{-1}\overset{\frown }{\mathrm{u}} $$

where $\widehat{\boldsymbol{a}}$ is the vector of SNP effects; $\overset{\frown }{\mathrm{u}}$ is vector of the GEBV obtained for the genotyped animals; ***W*** is a genotype matrix containing the numbers of reference alleles; ***D*** is a diagonal matrix of the weights of SNP variances, however, in this study the weights of SNP were not used, thus ***D*** = ***I*** (identity matrix). It is suggested that the use of SNP windows captures the QTL effects more efficiently than the use of a single SNP, and is relevant to distinguish effects from statistical noise [98]. Thus, the results of GWAS were reported as the proportion of variance explained by a window of 10 adjacent SNPs. The percentage (%) of genetic variance explained by each region was caling equation:

$$ \frac{Var\left({u}_i\right)}{\sigma_u^2}\times 100=\frac{Var\left({\sum}_{j=1}^{10}{W}_j{\widehat{a}}_j\right)}{\sigma_u^2}\times 100 $$

where *u*_*i*_ is the genetic value of the *i*-th region that consisted of 10 consecutive SNPs, ${\sigma}_u^2$ is the total genetic variance, *W*_*j*_ is the vector of gene content of the *j*-th SNP for all individuals, and ${\widehat{a}}_j$ is the marker effect of the *j*-th SNP within the *i*-th region.

### Searching for genes

Fatty acid profile traits are polygenic, being affected by many markers with small effect and non-genetic factors. Since the fatty acids traits evaluated in this study seem to be controlled mainly by many QTL of small effect, identifying large effect genes will be difficult, given each marker has a minor contribution to the total genetic variation. Consequently, only genomic regions explaining the largest proportion of additive genetic variation, above 0.30%, were considered to determine the possible QTL regions associated with fatty acids profile traits. The selected regions were used to identify positional candidate genes based on the starting and ending coordinates of each window by surveying the database available in the NCBI (National Center for Biotechnology Information) in OAR3.1 version of the ovine genome and Ensembl Genome Browser [99]. The description of genes regarding their biological function was performed by the DAVID [100] and BioGPS [101] online annotation databases. As needed, human genes were used as background in pathway and gene network investigation.

## Abbreviations

CLA: Conjugated linoleic acid
FA: Fatty acid
GEBV: Genomic breeding values
GO: Gene ontology
GWAS: Genome-wide association study
IMF: Intramuscular fat percentage
MUFA: Monounsaturated fatty acid
MUFAt: Total monounsaturated fatty acid
PCG: Putative candidate gene
PUFA: Polyunsaturated fatty acid
PUFAt: Total polyunsaturated fatty acid
QTL: Quantitative trait loci
SFA: Saturated fatty acid
SFAt: Total saturated fatty acid
SNP: Single nucleotide polymorphism
ω3t: Total of omega 3 fatty acids
ω6t: Total of omega 6 fatty acids
